# Coinfection with Epstein–Barr Virus (EBV), Human Papilloma Virus (HPV) and Polyoma BK Virus (BKPyV) in Laryngeal, Oropharyngeal and Oral Cavity Cancer

**DOI:** 10.3390/ijms18122752

**Published:** 2017-12-19

**Authors:** Bartłomiej Drop, Małgorzata Strycharz-Dudziak, Ewa Kliszczewska, Małgorzata Polz-Dacewicz

**Affiliations:** 1 Department of Information Technology and Medical Statistics, Medical University of Lublin, 20-059 Lublin, Poland; bartlomiej.drop@umlub.pl; 2 Chair and Department of Conservative Dentistry with Endodontics, Medical University of Lublin, 20-059 Lublin, Poland; 3 Department of Virology, Medical University of Lublin, 20-059 Lublin, Poland; ewakliszczewska@gmail.com (E.K.); m.polz@umlub.pl (M.P.-D.)

**Keywords:** squamous cell carcinoma (SCC), laryngeal cancer, oropharyngeal cancer, oral cancer, Epstein–Barr virus (EBV), human papillomavirus (HPV), BK virus (BKV), co-infection

## Abstract

Most research providing evidence for the role of oncogenic viruses in head and neck squamous cell carcinoma (SCC) development is focused on one type of virus without analyzing possible interactions between two or more types of viruses. The aim of this study was to analyse the prevalence of co-infection with human papillomavirus (HPV), Epstein–Barr virus (EBV) and polyoma BK virus (BKPyV) in oral, oropharyngeal and laryngeal squamous cell carcinomas in Polish patients. The correlations between viral infection, SCC, demographic parameters, evidence of metastases and grading were also investigated. Fresh-frozen tumour tissue samples were collected from 146 patients with laryngeal, oropharyngeal and oral cancer. After DNA extraction, the DNA of the studied viruses was detected using polymerase chain rection (PCR) assay. Males (87.7%) with a history of smoking (70.6%) and alcohol abuse (59.6%) prevailed in the studied group. Histological type G2 was recognized in 64.4% cases. The patients were most frequently diagnosed with T2 stage (36.3%) and with N1 stage (45.8%). Infection with at least two viruses was detected in 56.2% of patients. In this group, co-infection with HPV/EBV was identified in 34.1% of cases, EBV/BKV in 23.2%, HPV/BKV in 22.0%, and HPV/EBV/BKV in 20.7%. No difference of multiple infection in different locations of cancer was observed. The prevalence of poorly differentiated tumours (G3) was more frequent in co-infection with all three viruses than EBV or BKV alone. A significant correlation was observed between tumour dimensions (T) and lymph-node involvement (N) in co-infected patients compared to single infection. Further studies are necessary to clarify whether co-infection plays an important role in the initiation and/or progression of oncogenic transformation of oral, oropharyngeal and laryngeal epithelial cells.

## 1. Introduction

Head and neck cancer accounts for more than 550,000 cases and 380,000 deaths annually worldwide [1]. In Europe, in 2012, there were approximately 250,000 cases (an estimated 4% of cancer incidence) and 63,500 deaths due to malignancy in this area [2]. According to the Polish National Cancer Registry, oral and oropharyngeal cancer accounts for 3.8% cancers in men and 1.6% cancers in women, while laryngeal cancer constitutes 2.5% cancers in men and 0.4% in women [3].

It is estimated that approximately 90% of all head and neck cancers are squamous cell carcinoma (SCC). As many studies proved, the etiology of SCC is complex and involves many factors. Molecular and epidemiological research has provided evidence of the role of oncogenic viruses in SCC development [4,5,6,7]. Human papilloma virus (HPV) was established by the International Agency for Research on Cancer to be an important human carcinogen causing cancer in the head and neck area [8,9]. However, the first human virus with attributable oncogenic potential was Epstein–Barr Virus (EBV) [10]. The relationship between infection with EBV and the development of cancer in the head and neck region was reported by many researchers [11,12,13,14].

The human polyoma BK virus (BKPyV) belonging to the *Polyomaviridae* family is associated with human tumors and is classified in group 2B as possibly carcinogenic to humans [15,16,17]. It is estimated that 90% of the population may be infected with BK virus (BKV) during childhood. Moreover, BKV DNA has been found in many types of tumors, e.g., human brain tumors, in neuroblastoma, in urinary tract tumors, in uterine cervix vulva, lips and tongue carcinomas, as well as in Kaposi’s sarcoma [18,19,20,21]. The correlation between BKV and prostate and bladder carcinoma and between BKV and metastatic bladder carcinoma among immunosuppressed transplant recipients has been described as a result of possible BKV latency in the kidneys [22]. Another potential location of the virus are salivary glands, as BKV DNA has been detected in saliva [23]. Initial viral exposure often leads to latent infections. Latent episomal BK virus can be reactivated and it can then cause productive viral infections [24]. The etiologic contribution of BK Polyoma Virus (BKPyV) is suggested to represent mechanistically a ‘driver’ role to a given cancer [25].

In our previous study, BKPyV DNA was detected in 18.5% of patients with oral squamous cell carcinoma but only in 3.3% of the controls [26]. The present research investigated the prevalence of co-infection of human papillomavirus, Epstein–Barr virus and polyoma BK virus in fresh-frozen samples from patients with laryngeal, oropharyngeal and oral cavity cancer and analysed the effects of these co-infections on clinico-pathological and epidemiological features.

## 2. Results

Males (87.7%) with smoking (70.6%) and alcohol abuse (59.6%) problems prevailed in the studied group. A moderately differentiated (G2) histological type was recognized in 64.4% of cases. The patients were diagnosed most frequently with T2 stage (36.3%), and with N1 stage (45.8%) conditions. No distant metastasis was observed (M0 in 100% patients). Characteristics of patients with oral, oropharyngeal and laryngeal cancer are shown in Table 1.

Single infection was detected in 43.8% of patients and multiple infection in 56.2% (Table 2). Among 146 infected patients, HPV/EBV co-infection was observed in 34.1% of cases, HPV/BKV in 22.0% of cases, and EBV/BKV in 23.2%. Co-infection with all three viruses was detected in 17 cases (20.7%).

The relative frequencies of single infection with HPV, EBV and BKV among oral cavity, laryngeal and oropharyngeal cancers were significantly different (*p* = 0.0102). Single infection with HPV was most frequent in oral cavity cancer (44.5%), while EBV infection was most frequent in oropharyngeal cancer (57.1%) (Table 3). No difference of multiple infection in different locations was observed.

The frequency of co-infection was not dependent on the sex and age of the patients (Table 4). The relative frequency of HPV and EBV co-infection in males and females did not differ significantly from the relative frequency of each sex and age group in the study population. Co-infection was detected mainly in patients living in urban areas. No difference in the frequency of co-infection with the examined viruses in particular locations was found. In cases of co-infection, the histological grade G3 predominated (*p* < 10^−4^). Stages N1 and N2 were significantly more frequent in co-infection, while T1 and T2 were more frequent in co-infection with three types of viruses.

Logistic regression analysis showed a correlation between histological grade and T, N parameters and type of co-infection (Table 5). The prevalence of poorly differentiated tumours (G3) was more than four times more frequent in HPV/EBV co-infection (OR = 4.14; *p* = 0.0160), five times more frequent in EBV/BKV co-infection (OR = 5; *p* = 0.0080), and 10 times more frequent in patients co-infected with HPV/EBV/BKV (OR = 10.5; *p* = 0.0010) compared to infection with EBV alone.

Another significant relationship was found between histological grading and the type of virus while analyzing single BKV infection and co-infection with HPV/EBV/BKV. Poorly differentiated tumours (G3) were more than four times more common in patients co-infected with HPV/EBV/BKV (OR = 4.5; *p* = 0.0427) compared to infection with single BKV.

A significant correlation was observed between tumour dimensions (T) and lymph-node involvement (N) in co-infected patients compared with single infection. The T3–T4 stages were more frequent in co-infection of all three viruses than BKV alone (OR = 3.5; *p* = 0.0457). On the other hand, N3–N4 was detected more frequently in co-infection of all viruses than HPV alone (OR = 11.5; *p* = 0.0067), than EBV alone (OR = 8.8; *p* = 0.0160), and then BKV alone (OR = 14.4; *p* = 0.0204).

## 3. Discussion

Malignancies associated with infectious agents may result from prolonged latency as a consequence of chronic infections [27]. Pathogenic infections are necessary but not sufficient for cancer initiation or progression [8,10,17,25,27]. In patients infected with one virus, secondary co-infection with another virus may serve as an important co-factor that may cause initiation and/or progression of tumors.

A number of studies available in the literature concern the role of viruses in the development of head and neck squamous cell carcinoma (HNSCC). However, they are mainly focused on one type of virus, while only some recent reports analyze the possible correlation between the infection of two oncogenic viruses and carcinogenesis. Our research is the first original observation that implicates HPV, EBV, BKPyV co-infection in laryngeal, pharyngeal and oral cancer in the Polish population. The role of HPV virus, especially HPV16 in head and neck squamous cell carcinoma is well established [28,29,30,31,32,33,34,35,36,37,38].

In our study, a vast majority of patients co-infected with HPV/EBV smoked cigarettes. HPV/EBV coinfection was also detected statistically more often in patients who had problems with alcohol abuse. The results of many researchers suggest a possible synergy between tobacco components and viral oncogenes, especially HPV16 E6/E7 in transformation of oral epithelial cells [39,40,41].

The role of EBV in oral squamous cell carcinoma (OSCC) development was first observed by zur Hausen [42]. Other authors also emphasize the role of EBV in the development of OSCC [13,14,43,44]. Jaloluli et al. [13] detected the presence of EBV in 55% of samples from eight different countries. Primary infection with EBV mostly occurs at an early age. The virus, together with saliva, gets to the squamous epithelium and the lymphoid organs, primarily B-cells of the pharynx. There, the virus can survive in a latent form. EBV can reactivate periodically without symptoms and can be detected in the saliva of the infected patients [45].

A number of studies point to co-infection with HPV and EBV in oral squamous cell carcinoma [11,46,47]. Moreover, several researchers indicate that co-infection by multiple oncogenic viruses may be an important risk factor in the development of OSCC [11,42,47,48]. Co-infections occur much more frequently in the areas of high prevalence of infectious agents, especially in developing countries [27]. The presence of HPV/EBV co-infection in the presented study was found in 34.1% of patients. Deng et al. [49], in research carried out in Japan, revealed HPV/EBV co-infection in 1% of patients with head and neck cancer (HNC) and in 10% of patients with nasopharyngeal carcinoma (NPC).

Infection with a number of pathogens very often causes inflammation of tissues or organs, which can lead to the initiation of carcinogenesis. Al Moustafa et al. [46] proposes that high-risk HPV and EBV co-infections play an important role in initiating neoplastic transformation of human oral epithelial cells. Jiang et al. [47] hypothesized that oropharyngeal tumors might be associated with both HPV and EBV rather than HPV alone, and co-infected cells can have a higher tumorigenic potential than normal cells.

According to the research, it is not clear which virus, HPV or EBV, contributes to the first infection in co-infected patients [50]. However, the study performed by Makielski et al. [51] indicated that infection with HPV in the oral cavity may increase the capacity of epithelial cells to support the EBV life cycle, which could in turn increase EBV-mediated pathogenesis in the oral cavity. Guidry and Scott [52] suggested that HPV/EBV co-infection increases EBV persistence either through latency or enhanced viral replication and by extending HPV oncogene expression.

Apart from HPV and EBV, BKV DNA was also detected (20.7%) in the studied material. Recent data has suggested a correlation between BK virus and various types of human cancers [53].

The presence of BKV DNA was confirmed in high-grade squamous intraepithelial cervical lesions (precancerous lesions) [54]. Burger-Calderon et al. [55] suggest a connection between BKPyV and the oral cavity. Several studies have suggested BKPyV to be oral-tropic [23]. BKPyV binds to cellular receptors such as N-linked glycoprotein with a 2,3-linkedsialic acids and gangliosides GD1b and Gt1b, which is true for kidney (Vero) and oral (HSG) cells in vitro. It has not been determined whether the viruses undergo true latency, expressing only a subset of specific viral genes. It is unclear whether polyomavirus DNA is commonly integrated into the host genome or whether integration is a rare event specific to HPyV subtypes [17]. The non-coding control region (NCCR) is a hypervariable region and comparative studies suggested that it may regulate host cell tropism [55].

BKV DNA was detected in tonsilar biopsy specimens and nasopharyngeal aspirates. Moreover, replication of BKPyV laboratory strain in human submandibular and parotid salivary gland cell lines (HSG and HSY) was also demonstrated. Besides, Moens et al. [56] suggest that polyomaviruses, including those induced by other oncogenic viruses, may be a co-factor in the development of cancer. Some authors suggest that BK virus may be a potential co-factor for HPV in the development of cervical neoplasia [57], especially together with the HPV genotype 16 [54]. In our studies HPV/BKV co-infection was detected in 22.0% cases, while EBV/BKV in 23.2%. EBV/BKV was statistically more often detected among urban inhabitants. In light of our and other authors’ research results, we cannot exclude the role of BKV in SCC, considering the fact that the genetic material of BKV was detected in saliva [23]. The ‘hit and run’ hypothesis is a mechanism deemed valid to justify a co-factorial role of BKPyV in cancer onset and progression in humans [17]. Tag gene expression, leading to inactivation of p53, without evidence of a productive infection (i.e., viral protein expression, genome replication, etc.), leads to host cell transformation [58].

In our study the OR for low-differentiated tumours (G3) was about four times greater in patients with HPV/EBV co-infection, five times greater in patients with EBV/BKV co-infection, and more than 10 times greater in patients with HPV/EBV/BKV co-infection than in patients with only EBV infection. Gonzales-Moles et al. [59] found a correlation between EBV and poor differentiation of cancerous lesion in OSCC. Some researchers have revealed experimentally that EBV infection may delay epithelial differentiation and enhance the invasiveness of epithelial cells expressing *HPV16 E6* and *E7* oncogenes. However, delayed differentiation and greater invasiveness were still present in epithelial cells after loss of EBV, which may suggest that EBV infection led to epigenetic reprogramming [60].

Double or mixed infection with other oncogenic viruses may induce transformation. A limitation of our study is, however, only the epidemiological character of the research carried out. As DNA was extracted from resected tumour tissues, the sample might contain DNA from normal tissues, including infiltrating lymphocytes, which might be infected with viruses. Performing immunohistochemical analysis could have provide more conclusive data. Thus, further studies are needed to clarify whether BKPyV plays a role in oral squamous cell carcinoma or is a co-factor for cancers induced by other oncoviruses. It is well-known that chronic infection affects the immunological response of the host. Primary infection with a non-oncogenic virus may promote superinfection with an oncogenic virus capable of tumor transformation. The oncogenic potential of HPV is related to the expression of *E6* and *E7*, whereas the oncogenic potential of EBV to the expression of LMP-1 (latent membrane protein 1) and LMP-2 and of the BKV-LTag (large tumour antigen). These oncoproteins can be in cooperation and they can lead to the transformation of the oral epithelium [17,25,27]. Toll-like receptors (TLRs) play a critical role in the early innate immune response to invading pathogens by sensing microorganisms and they are involved in sensing endogenous danger signals. The LTag of the virus BKPyV as well as the protein of LMP-1 of the virus EBV lowers the expression of TLR9 [61,62]. EBV latent membrane protein 1 is a negative regulator of TLR9. These observations may contribute to future studies. There are few studies examining the association between HPV, EBV and/or BKV in the progression of oral cancers. A detailed understanding of co-infection will enable the targeting of new methods for the early detection, prevention and treatment of viral-associated cancers.

## 4. Materials and Methods

### 4.1. Patients

The present study involved 146 patients with a diagnosed and histopathologically confirmed SCC of larynx, oropharynx and the oral cavity who were infected with at least one virus—HPV, EBV, or BKV. The patients were hospitalized at the Otolaryngology Division of the Masovian Specialist Hospital in Radom, Poland. The patients had not received radiotherapy or chemotherapy before. The samples were collected during surgery, but TNM was calculated during primary diagnosis. TNM classification was done according to the criteria of the Union Against Cancer (UICC) [63]. Histological grading was performed according to World Health Organization criteria, which divide tumors into three types: well differentiated (G1), moderately differentiated (G2), and poorly differentiated (G3) [64].

The research was approved by the Medical University of Lublin Ethics Committee and is in accordance with the GCP regulations (no. KE-0254/133/2013, 23 May 2013).

### 4.2. DNA Extraction from Fresh Frozen Tumour Tissue; Detection of EBV DNA

DNA extraction from fresh frozen tumour tissue, detection of EBV DNA, and amplification of the *EBNA-2* gene (the nested PCR) were performed as previously described [65].

### 4.3. HPV Detection and Genotyping

HPV genotyping was performed using the INNO-LiPA HPV Genotyping Extraassay (Innogenetics, Gent, Belgium). The kit is based on the amplification of a 65 bp fragment from the L1 region of the HPV genome with a SPF10 primer set. PCR products are subsequently typed with the reverse hybridization assay. This kit identifies 28 HPV genotypes: HPV 6, 11, 16, 18, 26, 31, 33, 35, 39, 40, 43, 44, 45, 52, 52, 53, 54, 56, 58, 59, 66, 68, 69, 70,71, 73, 74, and 82.

### 4.4. Detection of BKV

The polymerase chain reaction (PCR) method was used to detect BKPyV in the specimens. With the aim of detecting the genetic material of the BKPyV, the primers described for the first time by Arthur et al. [66], namely PEP-1 (5′-AGTCTTTAGGGTCTTCTACC-3′) and PEP-2 (5′-GGTGCCAACCTATGGAACAG-3′) were used. The oligonucleotides attach to a highly conservative region of early coding T-Ag. Primers amplify a 176-bp fragment of BKV genetic material. The final concentrations of the PCR reaction mixture were as follows: 2.0 mM MgCl_2_, 200 µM dNTPs, 0.25 µM of each primer, 0.5 U Hot Start Taq DNA polymerase (Qiagen, Hilden, Germany). Amplification was performed under the following conditions: initial denaturation 94 °C 15 min, followed by 40 cycles: 94 °C 1 min, 55 °C 1 min, 72 °C 1 min; final extension: 72 °C 10 min. During each PCR run, the samples were tested, together with one negative and one positive control. DNA from the urine of a kidney transplant patient was used as a positive PCR control to assess the success of amplification (ATCC VR-837). PCR reagents without template DNA served as a negative control. The PCR products were analyzed using electrophoresis in 2% agarose gel.

### 4.5. Statistical Analysis

Statistical analysis was performed to investigate the relationship between the presence of HPV, EBV and BKV. The clinical and demographic characteristics of patients were determined by means of Pearson’s chi-square test and with Fisher’s exact test for small groups. Stepwise logistic regression was used to assess the effect of co-infection of HPV, EBV, BKV on the risk of the occurrence G and TN variables. The odds ratio with 95% confidence intervals was calculated. Statistical significance was defined as *p* < 0.05.

## 5. Conclusions

In Polish patients with oral, oropharyngeal and laryngeal cancer, co-infection with at least one virus was detected in 56.2% of cases. In this group, co-infection with HPV/EBV was identified in 34.1% of cases, EBV/BKV in 23.2%, HPV/BKV in 22.0%, and HPV/EBV/BKV in 20.7%. No difference of multiple infection in different locations of cancer was observed.

The prevalence of poorly differentiated tumours (G3) was more frequent in co-infection of all three viruses than EBV or BKV alone. T3–T4 and N3–N4 was more frequent in co-infection than in single viral infection.

Future epidemiological studies regarding the relationship between infection and gender, tobacco, alcohol and chronic inflammation in the development of oral cancer as well as studies on the mechanisms of co-infection and/or superinfection and their role in oral squamous cell carcinoma are necessary. Knowledge about the pathways of these viruses may provide targets for therapy and for devising diagnostic methods.

## Figures and Tables

**Table 1:** Epidemiological and clinical characteristics of patients.

		Total *n* = 146	
		*n*	%
Sex	Female	18	12.3
	Male	128	87.7
Age	<50	27	18.5
	50–69	92	63.0
	≥70	27	18.5
Place of residence	Urban	95	65.1
	Rural	51	34.9
Smoking	Yes	103	70.6
	No	43	29.4
Alcohol abuse	Yes	87	59.6
	No	59	40.4
Histological grading	G1	28	19.2
	G2	94	64.4
	G3	24	16.4
T stage	T1	29	19.9
	T2	53	36.3
	T3	27	18.5
	T4	37	25.3
N stage	N1	67	45.8
	N2	33	22.6
	N3	23	15.8
	N4	23	15.8
M stage	M0	146	100.0
	M1	0	0
Location of cancer	Oropharynx	53	36.3
	Larynx	40	27.4
	Oral cavity	53	36.3

T—tumour, dimensions; N—lymph nodes involvement; M—distant metastasis.

**Table 2:** Prevalence of human papillomavirus (HPV), Epstein–Barr virus (EBV) and BK virus (BKV) in infected patients.

**Single Infection**				
HPV	EBV		BKV	64 (43.8%)
18 (28.1%)	35 (54.7%)		11 (17.2)	
**Multiple Infection**				
HPV + EBV	HPV + BKV	EBV + BKV	HPV + EBV + BKV	82 (56.2%)
28 (34.1%)	18 (22%)	19 (23.2%)	17 (20.7%)	

**Table 3:** Prevalence of single and multiple infection according to location of cancer.

**Single Infection**							
**Location of Cancer**	**HPV**	**EBV**		**BKV**	**Total**		***p***
Oropharynx	4 (22.2%)	20 (57.1%)		4 (36.4%)	28	43.7%	0.0102 *
Larynx	6 (33.3%)	12 (34.3%)		4 (36.4%)	22	34.4%	
Oral cavity	8 (44.5%)	3 (8.6%)		3 (27.2%)	14	21.9%	
Total	18	35		11	64	100%	
**Multiple Infection**							
**Location of Cancer**	**HPV + EBV**	**HPV + BKV**	**EBV + BKV**	**HPV + EBV + BKV**	**Total**		***p***
Oropharynx	8 (28.6%)	8 (44.5%)	6 (31.6%)	3 (17.7%)	25	30.5%	0.7123
Larynx	6 (21.4%)	4 (22.2%)	4 (21.0%)	4 (23.5%)	18	21.9%	
Oral cavity	14 (50%)	6 (33.3%)	9 (47.4%)	10 (58.8%)	39	47.6%	
Total	28	18	19	17	82	100%	

* Statistically significant.

**Table 4:** Epidemiological and clinical characteristics of coinfected patients.

		HPV + EBV *n* = 28		HPV + BKV *n* = 18		EBV + BKV *n* = 19		HPV + EBV + BKV *n* = 17	
		*n*	%	*n*	%	*n*	%	*n*	%
Sex	female	2	7.1	2	11.1	2	10.5	1	5.9
	male	26	92.9	16	88.9	17	89.5	16	94.1
*p*		0.9030		0.9010		0.9402		0.6601	
Age	<50	4	14.3	2	11.1	3	15.8	1	5.9
	50–69	19	67.8	14	77.8	13	68.4	14	82.3
	≥70	5	17.9	2	11.1	3	15.8	2	11.8
*p*		0.9211		0.6831		0.9880		0.5910	
Place of residence	Urban	23	82.1	15	83.3	16	84.2	16	94.1
	Rural	5	17.9	3	16.7	3	15.8	1	5.9
*p*		0.0400 *		0.0640		0.0330 *		0.0440 *	
Smoking	Yes	22	78.6	13	72.2	15	78.9	13	76.5
	No	6	21.4	5	27.8	4	21.1	4	23.5
*p*		0.6010		0.7280		0.5691		0.7581	
Alcohol abuse	Yes	26	92.9	15	83.3	16	84.2	15	88.2
	No	2	7.1	3	16.7	3	15.8	2	11.8
*p*		0.0004 *		0.0890		0.0420 *		0.0800	
Histological grading	G1–G2	12	42.8	5	27.8	7	36.8	3	17.7
	G3	16	57.2	13	72.2	12	63.2	14	82.3
*p*		10^−4^ *		10^−4^ *		2 × 10^−4^ *		10^−4^ *	
T stage	T1–T2	20	71.4	14	77.8	13	68.4	15	88.2
	T3–T4	8	28.6	4	22.2	6	31.6	2	11.8
*p*		0.3310		0.4260		0.7180		0.0430 *	
N stage	N1–N2	22	78.6	16	88.9	16	84.2	17	100.0
	N3–N4	6	21.4	2	11.1	3	15.8	0	0
*p*		0.0349 *		0.0235 *		0.0274 *		0.0009 *	

* Statistically significant.

**Table 5:** Odds ratio of predictive variables.

	OR (95% CI)	OR (95% CI)	OR (95% CI)
**Variable**	**HPV/HPV + EBV**	**HPV/HPV + BKV**	**HPV/HPV + EBV + BKV**
G1–G2	0.8 (0.14–4.46)	0.61 (0.18–2.06)	0.4 (0.1–1.69)
G3	1.45 (0.54–3.87)	2.46 (0.73–8.25)	3.7 (0.88–15.27)
*p*	0.7580	0.2250	0.1440
T1–T2	0.91 (0.59–4.23)	1.7 (0.48–6.13)	2.46 (0.65–9.32)
T3–T4	1.57 (0.59–4.2)	0.6 (0.12–3.1)	0.44 (0.05–3.1)
*p*	0.7800	0.7950	0.4320
N1–N2	0.65 (0.21–2.12)	2.86 (0.81–10.24)	0.23 (0.03–1.93)
N3–N4	1.67 (0.03–2.25)	0.80 (0.08–7.79)	11.45 (1.37–95.6)
*p*	0.4330	0.4120	0.0067 *
**Variable**	**EBV/HPV + EBV**	**EBV/EBV + BKV**	**EBV/HPV + EBV + BKV**
G1–G2	0.54 (0.11–2.73)	0.40 (0.14–1.21)	0.19 (0.05–0.78)
G3	4.14 (1.52–11.23)	5.0 (1.61-15.54)	10.5 (2.49–44.11)
*p*	0.0160 *	0.0080 *	0.0010 *
T1–T2	1.12 (0.39–3.59)	1.78 (0.52–5.98)	3.04 (0.82–11.23)
T3–T4	0.61 (0.16–2.37)	0.91 (0.23–3.68)	0.31 (0.04–2.66)
*p*	0.8700	0.8280	0.3060
N1–N2	1.33 (0.52–3.42)	1.92 (0.61–6.09)	0.35 (0.04–2.96)
N3–N4	1.14 (0.27–4.84)	0.5 (0.06–4.36)	8.8 (1.07–72.34)
*p*	0.7060	0.6930	0.0160 *
**Variable**	**BKV/HPV + BKV**	**BKV/EBV + BKV**	**BKV/HPV + EBV + BKV**
G1–G2	0.38 (0.11–1.39)	0.53 (0.16–1.79)	0.25 (0.06–1.13)
G3	3 (0.82–10.9)	2.2 (0.64–7.19)	4.5 (1.01–20.1)
*p*	0.2160	0.3870	0.0427 *
T1–T2	2.5 (0.59–10.55)	2.1 (0.5–8.6)	1.43 (0.36–5.65)
T3–T4	0.36 (0.06–1.93)	0.8 (0.18–2.17)	3.5 (0.8–15.9)
*p*	0.4300	0.6780	0.0457 *
N1–N2	3.59 (0.9–13.9)	3.1 (0.88–11.61)	0.3 (0.03–2.74)
N3–N4	0.28 (0.03–2.62)	0.5 (0.09–2.99)	14.4 (1.6–126.1)
*p*	0.2920	0.8950	0.0204 *

* Statistically significant.
